# Network Pharmacology and Molecular Docking Study of Zhishi-Baizhu Herb Pair in the Treatment of Gastric Cancer

**DOI:** 10.1155/2021/2311486

**Published:** 2021-12-02

**Authors:** Ying Qu, Xiangyang Yang, Jingxiang Li, Shuxin Zhang, Shiying Li, Mengyuan Wang, Lu Zhou, Zhiying Wang, Zi Lin, Yuzhang Yin, Jinlong Liu, Nan Wang, Yang Yang

**Affiliations:** ^1^Dongzhimen Hospital, Beijing University of Chinese Medicine, Beijing 100700, China; ^2^School of Chinese Medicine, Beijing University of Chinese Medicine, Beijing 100029, China

## Abstract

**Objective:**

This study aimed to investigate the possible mechanism of the Zhishi and Baizhu herb pair in the treatment of gastric cancer by means of network pharmacology and molecular docking and to provide a theoretical basis for experiments and clinical application of traditional Chinese medicine for treating gastric cancer.

**Methods:**

The main active chemical components of Zhishi and Baizhu were screened through Traditional Chinese Medicine Systems Pharmacology (TCMSP) database and selected by using the thresholds of oral bioavailability ≥30% and drug-likeness ≥18%. The targets of Zhishi and Baizhu were obtained from TCMSP, Therapeutic Targets Database (TTD), and the DrugBank database. The corresponding genes of the targets were retrieved from the UniProt database, and the gastric cancer targets were obtained from the GeneCards database and TTD. Subsequently, the networks were built between the main drug components, drug targets, and gastric cancer targets. Then, the enrichment analyses of GO and KEGG were applied to predict the potential roles of gastric cancer pathogenesis via the R package clusterProfiler. Finally, molecular docking was used to determine the affinity between the targets and components.

**Results:**

Twenty-seven main active components were predicted from the Zhishi-Baizhu herb pair, and a total of 120 intersection genes were screened from 303 potential medicine genes and 1,839 disease genes. The enrichment included the PI3K-Akt and IL-17 signaling pathways, and the network analysis showed that the Zhishi-Baizhu herb pair acted on seven key targets, namely, AKT1, MMP9, IL-6, CCND1, BCL2, MTOR, and MDM2 (where they played a role in treating gastric cancer). Molecular docking showed that luteolin and naringenin could stably bind to the targets.

**Conclusion:**

The possible mechanisms of the components of the Zhishi-Baizhu herb pair in treating gastric cancer might be related to luteolin and naringenin, which intervened with the targets AKT1, MMP9, IL-6, CCND1, BCL2, MTOR, and MDM2, and are linked with the PI3K-Akt and IL-17 signaling pathways. This knowledge will lay a solid foundation for further experimental and clinical studies.

## 1. Introduction

Gastric cancer (GC) is a common malignant tumour in the world today and ranks third in fatalities and fifth in prevalence among malignant tumours [1]; it is the focus of clinical, epidemiology, and translational research at present [2].

Currently, surgical resection, perioperative adjuvant or neoadjuvant radiotherapy, immunotherapy, molecular targeted agents, and chemotherapy are the most effective treatments for this disease [3–5]. However, the poor prognosis at present may be due to the multiple-drug resistance of GC cells. Traditional Chinese medicine has been used to treat GC for thousands of years and is guided by a holistic concept, not only for local treatment but also for regulating the state of the whole body to control the development of a tumour [6, 7].

In recent years, the position of Chinese medicine in treating GC has also become increasingly crucial. As reported in previous studies, Chinese medicine can effectively inhibit the invasion and metastasis of GC cells and greatly improve the prognosis of GC patients, subsequently improving the quality of the lives of patients [8, 9].

In traditional Chinese medicine, GC belongs to the category of gastralgia, dysphagia, stomach reflux, abdominal mass, and others [10]. Its main pathogenesis is due to weakness of the spleen and stomach, phlegm and blood stasis, and deficiency-excess mixing. This leads to a disturbance of the circulation of qi, blood and body fluids, and an accumulation of water in the stomach. Additionally, toxins persist from stasis, which transform into cancer after a long time [11].

The herbal pairing of Zhishi (Aurantii Fructus Immaturus) and Baizhu (*Atractylodes macrocephala* Koidz.) has been frequently used for treating digestive diseases. This pairing was found by a famous Chinese physician named Zhang Zhongjing in the Eastern Han Dynasty [12], and, with its ideal curative effect, some later physicians developed its use in the treatment of GC. It also has a satisfactory clinical effect [13, 14].

Zhishi has a slightly cold nature (Chinese medicine theory) and a bitter, pungent, and acidic flavour, with a meridian tropism in the stomach and spleen. It is used for treating abdominal mass, dissipating phlegm, resolving mass issues, and settling stomach-Qi [15, 16]. As “Yao Pin Hua Yi” recorded, “Zhishi was especially used to remove the pathogenic factors of the stomach and to remove the agglomerate. The main therapeutic effect of Zhishi was in Zhongwan (epigastrium) to treat the blood level and to deal with the full pathogenic factors between umbilicus and stomach. It is also used to eliminate phlegm and remove the stagnation of pathogenic Qi in chest” [17]. Previous studies verified that flavonoids in Zhishi have anticancer effects and regulate cancer cell apoptosis and metastasis effectively by in vitro and in vivo experiments [18], and Yang Dong proved that sinensetin could inhibit the proliferation of human AGS GC cells and lead to cell cycle arrest in the G2/M phase and induce apoptosis [19].

Baizhu was first discovered in Shennong's Classic of Materia Medica as an upper grade drug; it has a warm nature and a bitter, sweet taste, with a meridian tropism in the stomach and spleen. It can invigorate the spleen and replenish Qi, induce diuresis, stop perspiration, dry dampness of the body, and calm a foetus [20]. The detailed description related to Baizhu was “dispelling dehumidification, harmonizing spleen and replenishing qi, strengthening the spleen and stomach, and promoting the appetite” in “Yi Xue Qi Yuan,” and “Changsha Yao Jie” said “Baizhu enters the Stomach Meridian of Foot-Yangming and the Spleen Meridian of Foot-Taiyin, with the use of dispelling dampness and causing dryness, invigorating spleen and replenishing qi, and so on. It can nourish stomach-Qi powerfully, reduce turbid Yin to promote the appetite, and it is good at preventing vomiting” [21]. Modern pharmacological studies have reported that Baizhu and its active sites have been shown to have anti-GC effects. Atractylenolide I can inhibit the growth and reproduction of GC MGC-803 cells, and atractylenolide II can induce apoptosis in the GC cell lines HGC-27 and AGS. Serum containing Baizhu can facilitate the apoptosis of GC cells line SGC-7901, side population cells, and nonside population cells. It can also inhibit the ability of cell proliferation and tumour formation. Furthermore, Baizhu can ameliorate the symptoms of patients with cachexia caused by GC and improve their appetite and body function state [22].

The compatibility of Zhishi and Baizhu is a classical prescription and the pairing had long been used to cure internal-injured spleen-stomach diseases [23], and the composition of these two drugs originated from the Zhi-Zhu decoction in the Synopsis of Golden Chamber. The Zhi-Zhu decoction consisted of seven Zhishi and two taels of Baizhu, which was mainly used to cure lumps under the heart. Many experiences have been summarised through the research of later generations of the physicians who used the derivate prescriptions of Zhi-Zhu decoction to prevent and treat GC in experiments or clinical studies and have achieved good therapeutic effects [24, 25]. The mechanism of these two herbs for treating GC is not clear at present. Hence, the aim of our research is to further investigate the detailed mechanism of the active components of Zhishi and Baizhu to treat GC systematically by using network pharmacology, thereby providing new theoretical basis for traditional Chinese medicine in the treatment of GC.

Network pharmacology and molecular docking are new technologies based on systems biology and database molecular correlation analysis for the exploration of new drugs and prediction of their related targets [26]. Network pharmacology has been used to explore the active components and potential targets relevant to the treatment of GC with Zhishi and Baizhu. Molecular docking was performed to verify the detailed interactions, mainly for affinities between Zhishi and Baizhu and the candidate targets. This research not only provides a comprehensive understanding of the molecular mechanism of Zhishi and Baizhu acting on GC but also offers a rapid and effective strategy for further experimental and clinical research in the future [27].

## 2. Materials and Methods

### 2.1. Main Component Screening

The main chemical compounds of the Zhishi (ZS) and Baizhu (BZ) herb pair were acquired from the Traditional Chinese Medicine Systems Pharmacology (TCMSP, https://tcmspw.com/tcmsp.php) database [28], and the main active chemical components were screened based on the threshold values of oral bioavailability (OB) ≥30% and drug-likeness (DL) ≥0.18, and the targets of the components were obtained from the TCMSP database and the Therapeutic Targets Database (TTD, https://bidd.nus.edu.sg/group/ttd/ttd.asp) [29] and combined with DrugBank (https://www.drugbank.ca) [30]. Subsequently, we input each of the candidate targets into the UniProt database (https://www.uniprot.org/) [31] to screen the human genes related to those targets (the species option was “*Homo sapiens*”), and the GC-related human genes were downloaded from the GeneCards database (https://www.genecards.org/) [32] and TTD using the keyword “GC.” Next, the candidate targets of the ZS-BZ herb pair against GC were obtained by taking an intersection of the above targets with a Venn diagram tool (https://bioinfogp.cnb.csic.es/tools/venny/index.html) [33].

### 2.2. Network Construction and Hub Gene Selection

We inputted these overlapped targets into STRING tools (https://string-db.org) [34] to construct the protein-protein interactions (PPI) network, and the required score was defaulted to be greater than 0.4. We selected “*Homo sapiens*” from species, and the compound-target-disease network was built and displayed using Cytoscape 3.6.1 software (https://cytoscape.org/) [35]. We used the cytoHubba plugin to select the key genes, and a degree greater than 2-fold above the median was used to evaluate and sequence the central genes. We chose the top 20 main related genes [36].

### 2.3. Gene Ontology and Kyoto Encyclopedia of Genes and Genomes Pathways Enrichment Analysis

Gene ontology (GO) and Kyoto Encyclopedia of Genes and Genomes (KEGG) enrichment analyses of potential targets were performed using the R package.

ClusterProfiler was used to search the related functions and pathways [37]; we set the following values: width = 35, height = 20, units = “cm,” and *P* value < 0.05 as the condition to visualise the results. Three selected analysis items were selected for GO enrichment analysis: molecular functions, cell components, and biological processes. Thus, we obtained the important terms of enriched GO terms and KEGG pathways to study the specific mechanism of the ZS-BZ herb pair against GC.

### 2.4. Molecular Docking Technology

The 3D structures of the target proteins were downloaded from the RCSB PDB database (https://www.rcsb.org/) [38]; the source of organism was set as “*Homo sapiens*” and refined at resolutions of 2.0–3.0 Å [26], and the active compounds were downloaded from TCMSP and were used as ligands. Docking results were analysed using PyMOL 2.3.0 to reveal the 3D protein-ligand complex, and we used AutoDockTools 1.5.6 software to remove water molecules [39, 40], add nonpolar hydrogen, and then calculate the affinities between these proteins and ligands. We carried out hydrogenation and charge calculation of these 20 main target proteins. The docking binding energy based on the threshold conditions of ≤ −5.0 kcal/mol was considered successfully to predict well compound-protein binding affinity [41], and the related conformations with good affinities were visualised in PyMOL 2.3.0.

## 3. Results

### 3.1. Targets of ZS-BZ Herb Pair against GC

**Figure 1:**
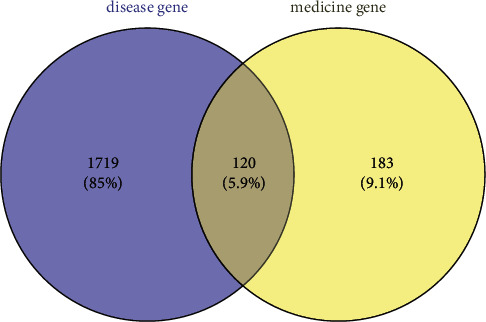
Venn diagram of the Zhishi-Baizhu (ZS-BZ) herb pair (medicine genes) and gastric cancer (disease genes) intersection targets. The 120 overlapping targets represent the candidate targets of the ZS-BZ herb pair against gastric cancer.

Excluding no known targets of compounds, a total of 27 main chemical compounds from the ZS (20) and BZ (7) herb pair were obtained from the TCMSP database, and 743 targets were retrieved from the TCMSP database, TTD, and DrugBank using the names of all the selected compounds above. The main compounds were luteolin, naringenin, 12-senecioyl-2E,8E,10E-atractylentriol, nobiletin, and tetramethoxyluteolin. The selected compounds are shown in Table 1. Then, we put these targets into the UniProt database and selected species option as “*Homo sapiens*”; a total of 303 human genes related to these medicine targets were screened out. In addition, a total of 1,839 GC-related targets were screened from the GeneCards database. All of the suitable herb targets are also shown in Table 1, and the predictive candidate targets of ZS and BZ overlapped with the GC-related targets. In total, 120 selected targets of ZS and BZ against GC were identified (excluding duplicate targets) and are shown in Figure 1.

### 3.2. Results of Network

#### 3.2.1. Results of PPI Network

**Figure 2:**
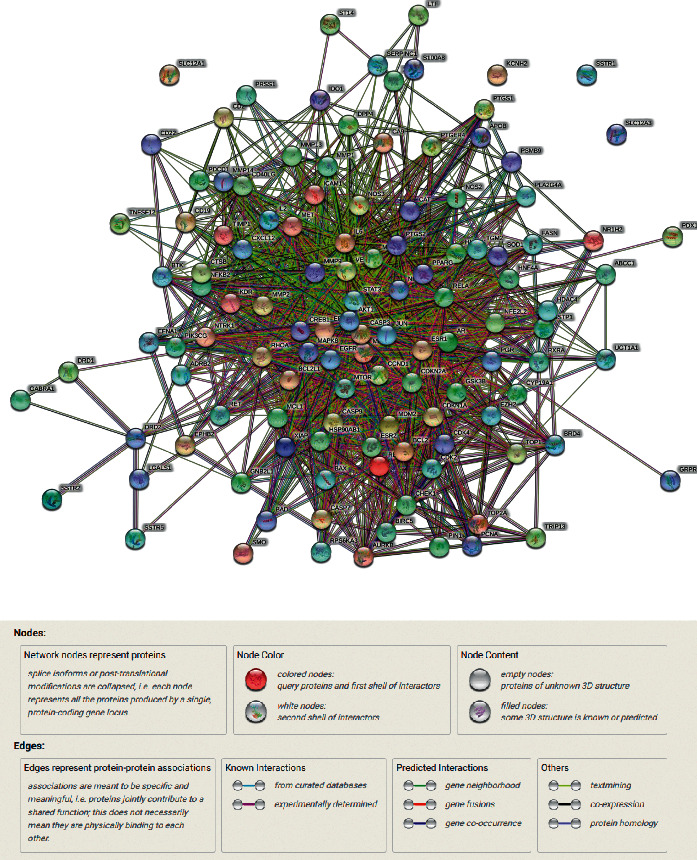
Protein-protein interaction networks of the Zhishi-Baizhu herb pair and gastric cancer.

The STRING tool was applied to construct the PPI network of the herb compound-disease-target relationships for the 120 overlapping targets. We selected a score >0.7 from the analysis. The network of PPI contained 120 nodes and 671 edges, the average node of degree was 11.2, and the average local clustering coefficient was 0.533 (as shown in Figure 2).

#### 3.2.2. Cytoscape Pharmacological Network Diagram of Targets and the Result of Hub Gene Selection

**Figure 3:**
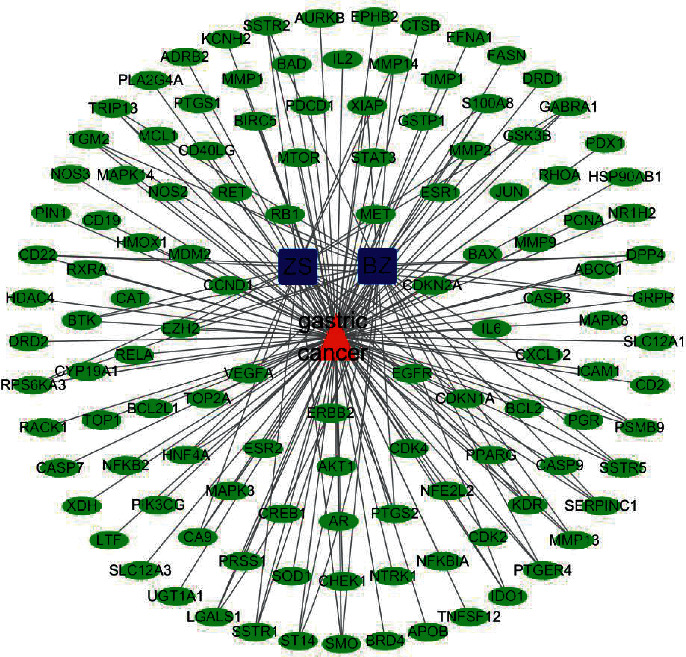
Drug-target interaction of the Zhishi-Baizhu (ZS-BZ) herb pair and gastric cancer. Red triangle represents gastric cancer, dark blue round rectangles represent the herbs ZS and BZ, and grass green ellipses represent potential common targets of the ZS-BZ herb pair and gastric cancer.

**Figure 4:**
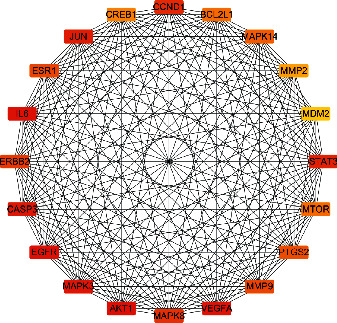
Top 20 key targets in PPI network.

We used Cytoscape 3.6.1 software to visualise the score targets of the results of the STRING database of the ZS-BZ herb pair and gastric cancer (Figure 3). Then we set the style of the node colour and size to construct drug-target interactions of the drug and disease. It contained 123 nodes and 178 edges, the average node degree was 11.2, and the top 20 important targets with an average degree value greater than 2 times of 11.2 were selected by the cytoHubba plugin. These top 20 targets were the key targets of the ZS-BZ herb pair against gastric cancer; the darker the colour, the higher the correlation (Figure 4 and Table 2).

### 3.3. Results of Enrichment Analysis

#### 3.3.1. GO Analysis

**Figure 5:**
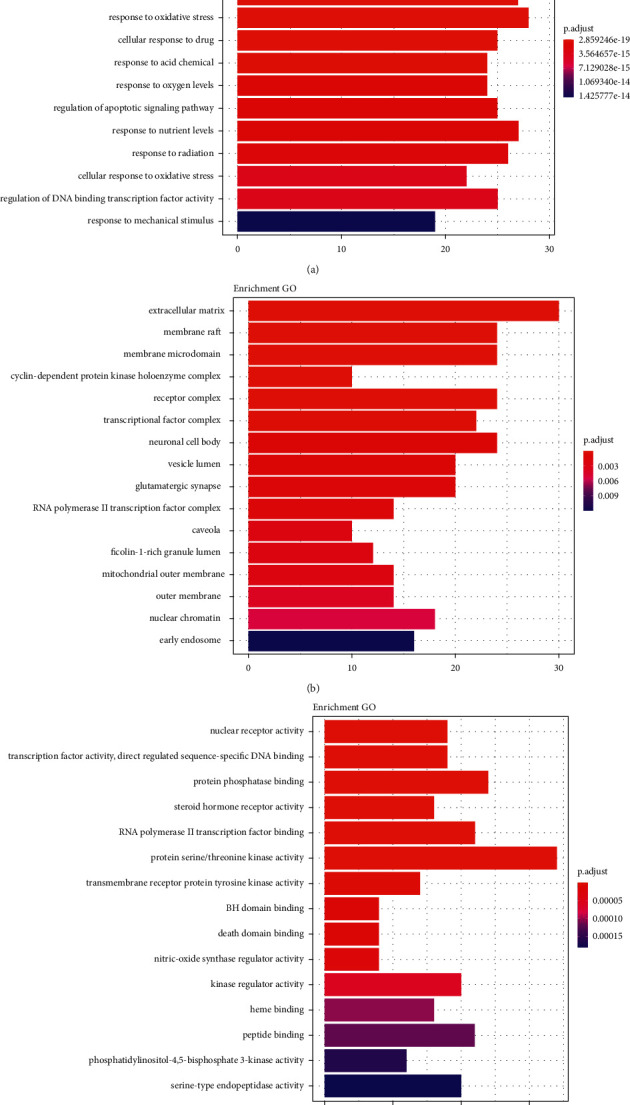
(a) Results of biological process (top 15). (b) Results of cellular components (top 16). (c) Results of molecular function (top 15).

The multiple mechanisms of the ZS-BZ herb pair against gastric cancer were identified using the R package clusterProfiler of Programming Language, and 46 enriched GO terms were identified (*P* < 0.05). The results of GO analysis were separated into three parts, as follows: (1) biological processes: response to lipopolysaccharide, cellular response to drugs, regulation of apoptotic signaling pathway, response to nutrient levels, and regulation of DNA-binding transcription factor activity (Figure 5(a)); (2) cellular components: membrane rafts, cyclin-dependent protein kinase holoenzyme complexes, membrane microdomain, receptor complexes, transcription factor complexes, and RNA polymerase II transcription factor complexes (Figure 5(b)); and (3) molecular functions: transcription factor activity, direct ligand-regulated sequence-specific DNA binding, protein phosphatase binding, and transmembrane receptor protein tyrosine kinase activity (Figure 5(c)).

#### 3.3.2. KEGG Analysis

**Figure 6:**
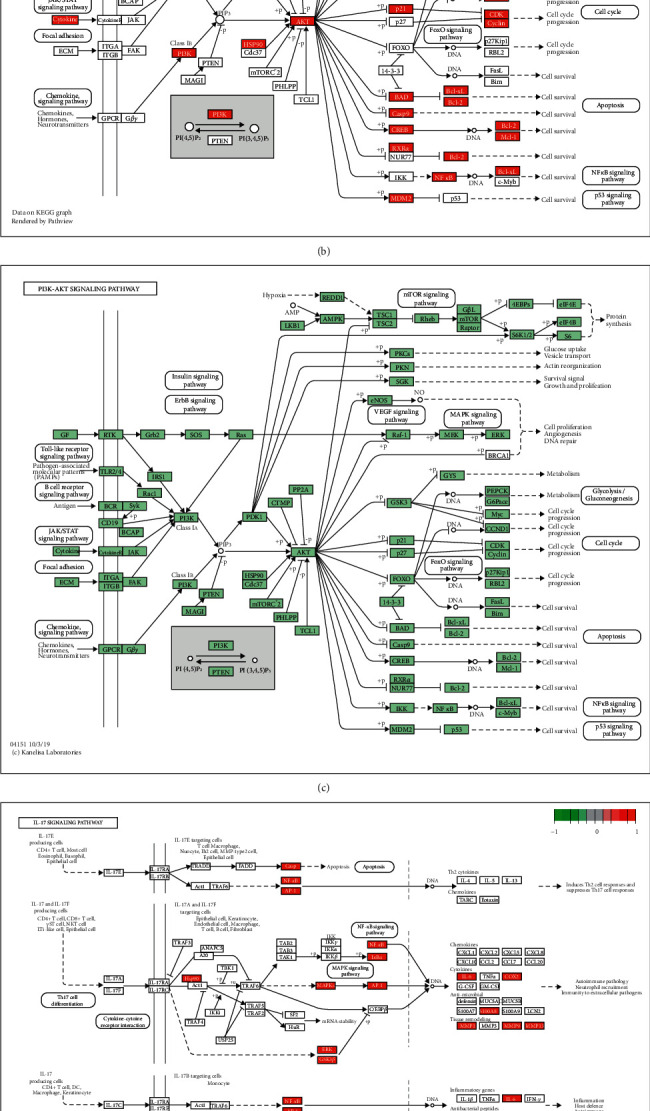
(a) Results of KEGG enrichment analysis (top 20). (b) KEGG pathway analysis of PI3K-Akt signaling pathway of the Zhishi-Baizhu herb pair in the regulation of gastric cancer. Red rectangles represent the targets of regulation roles. (c) KEGG pathway analysis of the PI3K-Akt signaling pathway of the Zhishi-Baizhu herb pair in the regulation of gastric cancer. Green rectangles represent the targets of potential roles. (d) KEGG pathway analysis of IL-17 signaling pathway of Zhishi-Baizhu herb pair in the regulation of gastric cancer. Red rectangles represent the targets of regulation roles. (e) KEGG pathway analysis of IL-17 signaling pathway of Zhishi-Baizhu herb pair in the regulation of gastric cancer. Green rectangles represent the targets of potential roles.

KEGG pathway enrichment of the top 20 pathways was identified (*P* < 0.05, Figure 6(a) and Table 3), including the PI3K-Akt and IL-17 signaling pathways, and the detailed targets of the regulatory roles of this pathway are presented in Figures 6(b)–6(e). The maps of the KEGG pathway showed that the most related targets of regulation roles were AKT, MMP9, IL-6, CCND1, BCL2, MTOR, and MDM2.

## 4. Results of Molecular Docking

**Figure 7:**
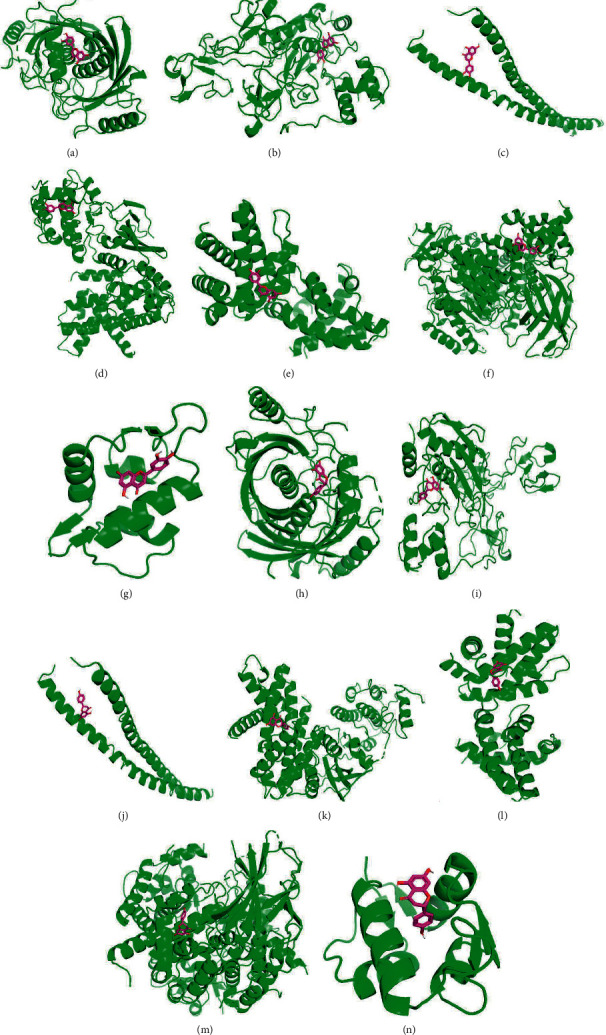
(a) Luteolin with AKT1. (b) Luteolin with MMP9. (c) Luteolin with IL-6. (d) Luteolin with CCND1. (e) Luteolin with BCL2. (f) Luteolin with MTOR. (g) Luteolin with MDM2. (h) Naringenin with AKT1. (i) Naringenin with MMP9. (j) Naringenin with IL-6. (k) Naringenin with CCND1. (l) Naringenin with BCL2. (m) Naringenin with MTOR. (n) Naringenin with MDM2.

We chose two main active compounds, luteolin and naringenin, which have a strong link with gastric cancer [42, 43], and molecular docking was used to verify the regulation of these two compounds in the top 20 selected target proteins. The results showed that most target proteins had a strong affinity for luteolin and naringenin. Except for CREB1 (PDB ID: 1ci6), the binding energies with luteolin and naringenin are −4.21 and −4.39 kcal/mol, respectively, whereas the other target proteins are all less than −5 kcal/mol. The molecular binding energies with luteolin and naringenin of the top 20 key target proteins are shown in Table 4, and the docking conformations of most related targets of regulation roles selected by KEGG analysis, such as AKT, MMP9, IL-6, CCND1, BCL2, MTOR, and MDM2, are shown in Figures 7(a)–7(n).

## 5. Discussion

Traditional Chinese medicine has been used in the struggle against diseases for thousands of years. The famous traditional Chinese physician Shi Jinmo found out many effects of the ZS-BZ herb pair for treating gastrointestinal diseases while studying drug pairs. ZS has been used to promote digestion, remove food, and resolve mass between belly and abdomen, whereas BZ has been used to harmonise qi and blood. When these two herbs are combined, one for eliminating and one for tonifying, as well as one for being fast and one for slowing down, they gain mutual restraint and utilisation with each other; thus, they finally achieve the therapeutic effect of tonifying without obstructing the stagnation (the theory of traditional Chinese medicine). They also relieve oppression and masses, eliminate pathogenic factors without damaging healthy qi, and strengthen the spleen and stomach [44].

Network pharmacology and molecular docking methods were used to explore the possible pharmacological mechanisms of the ZS-BZ herb pair related to gastric cancer in this study, which indicated that 27 compounds and 120 target genes were related to gastric cancer. According to the results of GO and KEGG pathway enrichment analyses, we found that the effects of the ZS-BZ herb pair against gastric cancer may be due to the active compounds of the ZS-BZ herb pair, especially for luteolin and naringenin which could influence the regulation of the PI3K-Akt and IL-17 signaling pathway. They may also be related to the apoptotic signaling pathway, cyclin-dependent protein kinase holoenzyme complex, and receptor complex and transcription factor complex. Based on the results of PPI analysis and Cytoscape Hub, AKT1, MMP9, IL-6, CCND1, BCL2, MTOR, and MDM2 were selected as key targets, and they reflect a strong affinity with luteolin and naringenin as the results of the molecular docking method.

Gastric mucosal carcinogenesis is a progressive process. The most recognized mode of intestinal gastric cancer is atrophic gastritis intestinal metaplasia dysplasia intramucosal cancer proposed by Correa [45] in 1988. The clinical symptoms of gastric cancer include epigastric pain, nausea, vomiting, anorexia, an epigastric lump, and the damage of gastric mucosal layer [46]. Recent studies have validated that these symptoms could effectively be ameliorated by the combination use of ZS and BZ. Zhizhu Pills can effectively regulate the expression of PI3K and Akt [47]. BZ can also repair gastrointestinal mucosal injury by regulating the expression of the IL-17 signaling pathway [48]; in our study, the PI3K-Akt and IL-17 signaling pathways are two key pathways involved in gastric cancer.

The PI3K-Akt signaling pathway is significant in the formation and progression of many malignancies, and it has become a hot spot as a therapeutic target for cancer. It involves the regulation of tumour cell of gastric cancer survival, proliferation, invasion, and migration [49, 50]. Various studies have indicated that the PI3K-AKT signaling pathway is associated with tumour resistance to chemotherapy and radiotherapy [51].

The experiments showed the overexpression of *AKT1* and *MTOR* genes in gastric mucosa of gastric cancer patients and showed that they play vital roles in cell growth, proliferation, metabolism, angiogenesis, and survival in mediating cellular functions [52]. Furthermore, mTOR is also involved in regulating the synthesis of biological macromolecules, such as nucleotides, proteins, and lipids. Another study showed that the metastasis of gastric cancer cells was induced by MMP9, and this process was activated by the PI3K-AKT signaling pathway. A further study found that the blockade of the PI3K-AKT signaling pathway by using MMP9 proenzyme inhibitor could suppress the distant metastasis of gastric cancer cells [53].

It has been reported that IL-6 is highly upregulated in many cancers and is one of the most important cytokine families in tumorigenesis and metastasis [54] and is highly expressed in gastric cancer tissues [55]. It can effectively control the pathological changes of cancer by mediating the expression of IL-6 in the IL-17 signaling pathway [56]. In addition, CCND1 is an important target for cell proliferation in gastric cancer [57], and it has been shown that high CCND1 expression levels are closely related to shorter survival rates of gastric cancer patients. It has been found in tissues of gastric cancer that the upregulated expression of BCL2 predicted a poor prognosis [58].

Some scientific tests have established that luteolin is an active component of ZS, which is a potential chemotherapeutic agent against gastric cancer. It can suppress cell cycle arrest, proliferation, colony formation, and accelerate apoptosis in gastric cancer cells in vitro and in vivo by regulating the PI3K and AKT signaling pathways [59]. In addition, studies have substantiated that luteolin can significantly inhibit the IL-17 signaling pathway in digestive tract disease [60] and then decrease the level of IL-6 in the patient's serum.

Naringenin induces apoptosis of gastric cancer SGC7901 cells by decreasing the expression of MMP9 by downregulating the AKT pathway [61]. It can promote the apoptosis of human gastric cancer SGC7901 cells by inhibiting the PI3K-Akt signaling pathway and plays a tumour suppressor effect, providing a proper therapeutic target for the treatment of gastric cancer [62]. In our study, we preliminarily explored the mechanism of the ZS-BZ herb pair in treating gastric cancer by using network pharmacology and molecular docking. We found that there is a close link with the regulation of the PI3K-Akt and IL-17 signaling pathway with luteolin and naringenin. AKT1, MMP9, IL-6, CCND1, BCL2, MTOR, and MDM2 were found to be the seven key targets in these two signaling pathways, and they reflect a strong affinity with luteolin and naringenin.

## 6. Conclusions

In summary, the results of our study showed that the ZS-BZ herb pair may play a therapeutic role in treating gastric cancer based on the active compounds luteolin and naringenin. These two compounds mainly act on seven targets, namely, AKT1, MMP9, IL-6, CCND1, BCL2, MTOR, and MDM2; they may be related to the regulation of the PI3K-Akt signaling pathway and IL-17 signaling pathway. Our efforts have provided evidence and we have obtained a good understanding of the multitarget, multicompound, and multipathway synergy of traditional Chinese medicine in treating gastric cancer. However, in vivo and in vitro experiments and clinical studies should be conducted to verify the mechanism of the ZS-BZ herb pair against gastric cancer.

## Figures and Tables

**Table 1:** The information of 743 candidate compounds in the Zhishi-Baizhu herb pair.

Herb	Mol ID	Molecule name	OB (%)	DL	Target amount
ZS	MOL013277	Isosinensetin	51.15	0.44	27
ZS	MOL013279	5,7,4′-Trimethylapigenin	39.83	0.3	16
ZS	MOL013428	Isosakuranetin-7-rutinoside	41.24	0.72	1
ZS	MOL013430	Prangenin	43.6	0.29	3
ZS	MOL013433	Prangenin hydrate	72.63	0.29	1
ZS	MOL013435	Poncimarin	63.62	0.35	4
ZS	MOL013436	Isoponcimarin	63.28	0.31	4
ZS	MOL013437	6-Methoxyaurapten	31.24	0.3	11
ZS	MOL013440	Citrusin B	40.8	0.71	1
ZS	MOL001798	Neohesperidin_qt	71.17	0.27	7
ZS	MOL001803	Sinensetin	50.56	0.45	21
ZS	MOL001941	Ammidin	34.55	0.22	8
ZS	MOL002914	Eriodyctiol (flavanone)	41.35	0.24	8
ZS	MOL004328	Naringenin	59.29	0.21	37
ZS	MOL005100	5,7-Dihydroxy-2-(3-hydroxy-4-methoxyphenyl)chroman-4-one	47.74	0.27	10
ZS	MOL005828	Nobiletin	61.67	0.52	35
ZS	MOL005849	Didymin	38.55	0.24	13
ZS	MOL000006	Luteolin	36.16	0.25	57
ZS	MOL007879	Tetramethoxyluteolin	43.68	0.37	32
ZS	MOL009053	4-[(2S,3R)-5-[(E)-3-Hydroxyprop-1-enyl]-7-methoxy-3-methylol-2,3-dihydrobenzofuran-2-yl]-2-methoxy-phenol	50.76	0.39	11
BZ	MOL000020	12-Senecioyl-2E,8E,10E-atractylentriol	62.4	0.22	184
BZ	MOL000021	14-Acetyl-12-senecioyl-2E,8E,10E-atractylentriol	60.31	0.31	114
BZ	MOL000022	14-Acetyl-12-senecioyl-2E,8Z,10E-atractylentriol	63.37	0.3	115
BZ	MOL000028	*α*-Amyrin	39.51	0.76	1
BZ	MOL000033	(3S,8S,9S,10R,13R,14S,17R)-10,13-Dimethyl-17-[(2R,5S)-5-propan-2-yloctan-2-yl]-2,3,4,7,8,9,11,12,14,15,16,17-dodecahydro-1H-cyclopenta[a]phenanthren-3-ol	36.23	0.78	1
BZ	MOL000049	3*β*-Acetoxyatractylone	54.07	0.22	16
BZ	MOL000072	8*β*-Ethoxyatractylenolide III	35.95	0.21	5

**Table 2:** Key targets in protein-protein interaction network which are greater than the average value (top 20).

Rank	Name	Degree
1	AKT1	85
2	EGFR	75
2	IL-6	75
4	VEGFA	74
5	MAPK3	73
6	CASP3	72
7	JUN	70
8	STAT3	69
9	CCND1	67
10	MAPK8	66
11	ERBB2	61
12	ESR1	60
12	MMP9	60
14	PTGS2	58
15	MAPK14	56
16	BCL2L1	53
16	MTOR	53
18	CREB1	50
19	MMP2	48
20	MDM2	47

**Table 3:** Results of KEGG enrichment analysis (top 20).

ID	Description	*P* value	*P* adjusted	Count
hsa05215	Prostate cancer	4.71*E*-20	7.21*E*-18	21
hsa01522	Endocrine resistance	5.93*E*-20	7.21*E*-18	21
hsa05212	Pancreatic cancer	4.76*E*-18	3.85*E*-16	18
hsa05163	Human cytomegalovirus infection	1.99*E*-16	1.16*E*-14	25
hsa04151	PI3K-Akt signalling pathway	2.39*E*-16	1.16*E*-14	30
hsa05167	Kaposi's sarcoma-associated herpesvirus infection	7.92*E*-16	3.21*E*-14	23
hsa05223	Non-small cell lung cancer	1.15*E*-15	4.00*E*-14	16
hsa01524	Platinum drug resistance	1.46*E*-15	4.44*E*-14	16
hsa05161	Hepatitis B	2.96*E*-15	7.99*E*-14	21
hsa05222	Small cell lung cancer	3.78*E*-15	8.53*E*-14	17
hsa05219	Bladder cancer	3.86*E*-15	8.53*E*-14	13
hsa04933	AGE-RAGE signaling pathway in diabetic complications	1.63*E*-14	3.30*E*-13	17
hsa05210	Colorectal cancer	2.32*E*-14	4.26*E*-13	16
hsa05169	Epstein-Barr virus infection	2.46*E*-14	4.26*E*-13	22
hsa05162	Measles	2.63*E*-14	4.26*E*-13	19
hsa01521	EGFR tyrosine kinase inhibitor resistance	1.16*E*-13	1.75*E*-12	15
hsa04210	Apoptosis	2.38*E*-13	3.41*E*-12	18
hsa05166	Human T-cell leukaemia virus 1 infection	1.29*E*-12	1.74*E*-11	21
hsa04657	IL-17 signaling pathway	1.68*E*-12	2.07*E*-11	15
hsa05145	Toxoplasmosis	1.70*E*-12	2.07*E*-11	16

**Table 4:** The binding energies of top 20 key target proteins with luteolin and naringenin.

Target	PDB ID	Luteolin (kcal/mol)	Naringenin (kcal/mol)
AKT1	4gah	−7.45	−6.18
EGFR	4qvx	−6.42	−5.83
IL-6	6 mg^3^	−6.86	−5.51
VEGFA	6v7k	−6.03	−6.63
MAPK3	4qtb	−6.52	−5.2
CASP3	4jqy	−6.16	−6.07
JUN	3v3v	−6.2	−6.06
STAT3	6smb	−7.01	−5.05
CCND1	2w9f	−6.84	−5.86
MAPK8	3o2m	−5.77	−5.04
ERBB2	4hrn	−5.1	−5.41
ESR1	1a52	−5.42	−5.77
MMP9	1l6j	−6.78	−6.45
PTGS2	5ikr	−6.36	−5.8
MAPK14	5omh	−7.03	−6.77
BCL2L1	4qvx	−7.59	−7.41
MTOR	3qar	−6.36	−5.35
CREB1	1ci6	−4.21	−4.39
MMP2	1rtg	−6.67	−6.06
MDM2	3lbk	−5.78	−5.79

## Data Availability

All the data and materials used in the current study are available from the corresponding author upon reasonable request.
